# Pterostilbene-Isothiocyanate Conjugate Suppresses Growth of Prostate Cancer Cells Irrespective of Androgen Receptor Status

**DOI:** 10.1371/journal.pone.0093335

**Published:** 2014-04-03

**Authors:** Kumar Nikhil, Shruti Sharan, Ajanta Chakraborty, Partha Roy

**Affiliations:** Molecular Endocrinology Laboratory, Department of Biotechnology, Indian Institute of Technology Roorkee, Roorkee, Uttarakhand, India; National Health Research Institutes, Taiwan

## Abstract

Chemotherapy and anti-hormonal therapies are the most common treatments for non-organ-confined prostate cancer (PCa). However, the effectiveness of these therapies is limited, thus necessitating the development of alternative approaches. The present study focused on analyzing the role of pterostilbene (PTER)-isothiocyanate (ITC) conjugate – a novel class of hybrid compound synthesized by appending an ITC moiety on PTER backbone – in regulating the functions of androgen receptor (AR), thereby causing apoptosis of PCa cells. The conjugate molecule caused 50% growth inhibition (IC_50_) at 40±1.12 and 45±1.50 μM in AR positive (LNCaP) and negative (PC-3) cells, respectively. The reduced proliferation of PC-3 as well as LNCaP cells by conjugate correlated with accumulation of cells in G2/M phase and induction of caspase dependent apoptosis. Both PI3K/Akt and MAPK/ERK pathways played an important and differential role in conjugate-induced apoptosis of these PCa cells. While the inhibitor of Akt (A6730) or Akt-specific small interference RNA (siRNA) greatly sensitized PC-3 cells to conjugate-induced apoptosis, on the contrary, apoptosis was accelerated by inhibition of ERK (by PD98059 or ERK siRNA) in case of LNCaP cells, both ultimately culminating in the expression of cleaved caspase-3 protein. Moreover, anti-androgenic activity of the conjugate was mediated by decreased expression of AR and its co-activators (SRC-1, GRIP-1), thus interfering in their interactions with AR. All these data suggests that conjugate-induced inhibition of cell proliferation and induction of apoptosis are partly mediated by the down regulation of AR, Akt, and ERK signaling. These observations provide a rationale for devising novel therapeutic approaches for treating PCa by using conjugate alone or in combination with other therapeutics.

## Introduction

Despite significant efforts made towards the ablation of cancers, prostate cancer (PCa) is the most frequently diagnosed cancer and the second leading cause of cancer death among men in the United States, with an estimated 217,730 new cases and 32,050 deaths in 2010 [1]. Although the etiology of PCa remains unknown, elevated levels of steroid hormones, such as androgens and estrogens, as well as growth factors, such as insulin-like growth factor 1, are considered to be important risk factors [2]–[4]. Androgen ablation therapy has an initial response, but most patients with advanced PCa eventually develop resistance to this therapy and progresses to hormone-refractory prostate cancer (HRPC), for which there is no curative therapy [5]. Lack of effective treatment options for the management of HRPC reinforce the necessity to develop novel compounds that act singly or in combination.

Androgen and AR functions play a pivotal role in carcinogenesis and progression of PCa, as well as in normal prostate development [6], [7]. The actions of androgens, such as testosterone and dihydrotestosterone (DHT) are mediated by AR, which is a member of the nuclear receptor super family of ligand-dependent transcription factors [8]. In addition to androgen, AR activity may also be modified by molecules in other cell signaling pathways. Up regulation of epidermal growth factor receptor (EGFR) and subsequent increases in extracellular-regulated kinase (ERK) and Akt signaling, are implicated in PCa progression [9]. Akt regulates the AR signaling pathway by phosphorylation and/or transcriptional regulation of AR. Akt phosphorylates AR at serines 210/213 and 790/791 and finally transactivates its activity. An earlier study showed that inhibition of Akt pathway abrogates the HER-2/neu-induced AR signaling activity [10]. These results suggest that Akt is an activator of AR required for androgen-independent survival and growth of PCa cells. Research has shown that inhibition of one or both of these pathways has a more profound effect on tumor cell development and death, making them attractive combinational targets in PCa therapy. Therefore, AR, Akt, and ERK could be potential targets for the treatment of PCa.

Bioactive food components, in particular, are increasingly being evaluated as potential PCa chemopreventive agents because of their presumed safety [11]. One such compound is pterostilbene (PTER), a naturally occurring dimethyl ether analogue of resveratrol (RESV), which has higher oral bioavailability and enhanced potency as compared to RESV [12]. Several studies have shown that PTER can inhibit the growth of various hormone-responsive cancers, such as breast [13]–[15] and PCa [14], [16]–[18] both *in vitro* and *in vivo*. Although the anti-metastatic, anti-tumor and anti-leukemic properties of PTER have been well established in breast cancer, there are limited reports on the action of PTER in the proliferation of PCa cells induced by steroid hormones [16]. Similarly, isothiocyanates (ITCs) are naturally occurring, low molecular weight organic compounds with the general formula R-NCS [19]. These chemoprotective agents are found in a wide variety of cruciferous vegetables like broccoli, cauliflower, cabbage, brussels sprout, and kale [20]. Epidemiological studies have suggested that increased consumption of cruciferous vegetables may be protective against PCa risk [21]–[24]. However, in spite of compelling epidemiological correlation, the activity of ITCs against PCa cells is yet to be systematically assessed. Since the individual roles of PTER and ITC have already been implicated in PCa, we intended to develop a conjugate of PTER-ITC in search of potent anti-cancer molecules. The conjugate was prepared by appending ITC containing thiosemicarbazide pharmacophore with PTER backbone as described earlier [25]. Surprisingly, the conjugate molecule when tested in vitro, showed effective cytotoxicity in wide range of cancer cell lines at comparatively lower dose as compared to its parent compound i.e., PTER [25]. Further, our study indicated that the anti-proliferative activity of conjugate against human breast cancer cell lines was due to its ability to arrest cells in G2/M phase and activation of caspase, and also correlated with the blockade of Akt and ERK signaling pathways [25].

In the present study we examined detailed efficacy and molecular mechanisms of action of PTER-ITC conjugate using androgen-dependent (LNCaP) and androgen-independent (PC-3) PCa cells. Further, we also compared the efficacy of PTER-ITC conjugate with the parent compound of PTER i.e., RESV. Our study thus identified the newly developed conjugate to be a potent AR-inhibitor that strongly attenuates the growth of PCa cells in vitro by modulating AR expression as well as regulating cell cycle progression and apoptosis.

## Materials and Methods

### Reagents

**Figure 1:**
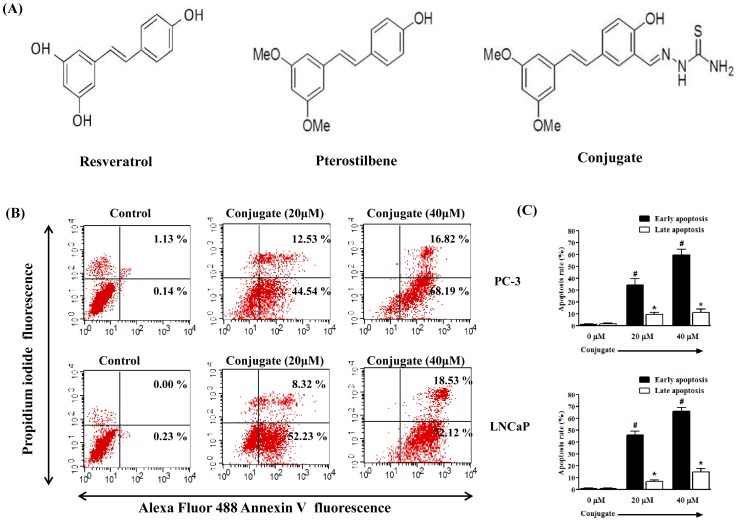
Induction of apoptosis by conjugate in prostate cancer cell line. (A) Structure of Resveratrol, Pterostilbene and Pterostilbene-isothiocyanate conjugate. (B) The effect of conjugate on the apoptosis of PC-3 and LNCaP cells as demonstrated by a representative FACS analysis using Annexin V as marker. (C) The histogram showing the data for FACS analysis where the results are the mean ± SEM of three independent experiments. # and *represents statistically significant difference with respect to their specific controls (vehicle treated) for cells in early and late apoptosis respectively at *p*<0.05.

All cell culture reagents were obtained from GIBCO (Invitrogen, CA, USA), unless otherwise stated. Penicillin, streptomycin, MTT (3-(4, 5-dimethyl-2-thiazolyl) 2,5diphenyl-2H-tetrazoliumbromide), cell culture grade dimethyl sulphoxide (DMSO), agarose and all analytical grade chemicals were from HiMedia (Mumbai, India). Reverse transcription- polymerase chain reaction (RT-PCR) kits were from Genei (Bangalore, India). RESV, PTER, DHT, Akt1/2 kinase inhibitor, PD98059 (ERK inhibitor), Z-VAD-FMK (pan caspase inhibitor), Z-LEHD-FMK (caspase-9 specific inhibitor), Z-IETD-FMK (caspase-8 specific inhibitor) and BCA protein estimation kits were from Sigma-Aldrich (St. Louis, MO, USA). Polyfect transfection reagent was purchased from QIAGEN (Valencia, CA, USA). Pifithrin-α (p53 inhibitor), antibodies for caspase-3, Bax, Akt, p-Akt, ERK, p-ERK, SRC-1, GRIP-1, N-CoR, β-actin and small interfering RNAs (siRNAs) against Akt (sc-43609), ERK (sc-35335) and control (sc-37007; negative control for experiments using targeted siRNA transfection; each consists of a scrambled sequence that will not lead to the specific degradation of any known cellular mRNA) were purchased from Santa Cruz Biotechnology (Santa Cruz, CA, USA). PTER-ITC conjugate was synthesized in the asymmetric synthesis laboratory of the Department of Chemistry, Indian Institute of Technology Roorkee, India, according to the procedure described earlier [25] and henceforth designated as conjugate in the manuscript (Fig. 1A).

### Cell Lines and Culture

The prostate carcinoma cell lines (AR-positive LNCaP and AR-negative PC-3) and noncancer cell lines (CHO and COS-1) were obtained from the National Centre for Cell Science (NCCS), Pune, India. PC-3, CHO and COS-1 cells were maintained in Dulbecco’s modified Eagle’s media (DMEM) while LNCaP cells were maintained in RPMI-1640 medium supplemented with 10% foetal bovine serum (FBS) (heat inactivated) under 5% CO_2_ at 37°C. All the experiments were performed using LNCaP, PC-3, CHO and COS-1 cells from passage below 29, 34, 20 and 30 respectively. The cells were washed properly before changing the media to steroid-free complete media prior to each treatment with the compounds, unless otherwise stated.

### Cytotoxicity Assays

MTT assay was carried out as described previously [25]. In brief, 5×103 cells in 200 μl of medium were seeded in 96-well plates (Griener, Germany*).* After 24 h, the cells were treated with various concentrations (0.1, 1, 10, 100 and 1000 μM) of RESV, PTER and conjugate. The control cells were treated with 0.1% DMSO (vehicle control). The cultured cells were assayed after 24 h by adding 20 μl of 5 mg/ml MTT followed by incubating at 37°C for 4 h. The MTT containing media was then aspirated and 200 μl DMSO (Himedia, Mumbai, India) was added to dissolve the formazone crystals. The optical density (OD) was measured at 570 nm using ELISA plate reader (Fluostar optima, BMG Labtech, Germany). The percentage inhibition was calculated as:
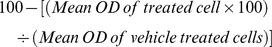



The dose response curve and IC_50_ values were obtained by nonlinear regression analysis [non-linear regression (sigmoidal dose response with variable slope)] using Graph Pad Prism, version 5.02 software (Graph Pad Software Inc., CA, USA).

### Cell Cycle Distribution and Apoptosis Assay by Flow Cytometry

Cell cycle distribution and Annexin V/Propidium iodide (PI) positive cells were analyzed using flow cytometry. In brief, first the cells were seeded and treated with 0, 10, 20 and 40 μM conjugate in complete medium for 24 h. This was followed by trypsinizing and fixing in 70% ethanol, and finally washing with PBS. Subsequently, the cells were treated with RNase A (50 μg/ml), stained with PI (50 μg/ml) and incubated in the dark for 30 min at room temperature and analyzed by flow cytometry for cell cycle distribution. For apoptosis, the conjugate treated cells were stained with Alexa-Fluor 488-conjugated Annexin-V using the Vybrant-Apoptosis Assay Kit from Invitrogen (USA) as per the manufacturer’s protocol. The stained cells were then analyzed by fluorescence activated cell sorting (FACS Calibur, BD Biosciences, San Jose, CA, USA) and the data were standardized using Cell Quest 3.3 software.

### Caspase Assay

Caspase activity was determined using ApoTarget caspase colorimetric protease assay sampler kit (KHZ1001; Invitrogen) according to the manufacturer’s instructions. Briefly, both the PCa cells were treated with increasing doses of conjugate and RESV for 24 h. The cells were then collected, washed in PBS, and lysed in 50 μl lysis buffer on ice for 10 min. After centrifugation, the supernatant containing 150 μg proteins were incubated with 200 μM of caspase-3 (Ac-DEVD-pNA), caspase-8 (Ac-IETD-pNA) and caspase-9 (Ac-LEHD-pNA) substrates respectively in reaction buffer at 37°C for 1 h in 96 well flat bottom plate. Levels of released pNA were then measured at 405 nm wavelength with ELISA plate reader (Fluostar optima, BMG Labtech, Germany). The fold-increase in caspase-3, -8, and -9 activities were determined by direct comparison to the level of un-induced control. For the caspase inhibitor assay, cells were pretreated with a synthetic pan-caspase inhibitor (Z-VAD-FMK), caspase-8 inhibitor (Z-IETD-FMK) and caspase-9 inhibitor (Z-LEHD-FMK) for 1 h before the addition of conjugate and the cell death were analyzed by MTT assay as discussed earlier.

### RT-PCR Analysis

Total RNA was extracted from the treated cells using RNA isolation kit obtained from Genei (Bangalore, India). The extracted RNA samples were then quantified and equal amount of the individual treatments was transcribed with the help of an RT - PCR kit from Genei (Bangalore, India) according to the manufacturer’s instruction. PCR was performed by denaturing at 94°C for 60 s, annealing at various temperatures (depending on primer pairs used) for 45 s and extension at 72°C for 2 min followed by the number of cycles for amplification. Primers for Bcl-2, Bcl-xL, Bax, AR and β-Actin were designed with the help of Primer 3 software and standardized in the laboratory. The PCR products were then separated on 2% agarose gel and visualized in a gel documentation system (Bio Rad, USA). The intensity of the bands on agarose gels were analyzed using ImageJ 1.43 software (NIH, USA) and normalized with respect to β-actin PCR products. Each of the RT-PCR was carried out three times. Table S1 presents the primer sequence, product size, annealing temperature, and number of cycles used for all primers.

### Immunofluorescence Staining

For immunofluorescence staining, LNCaP cells were washed with PBS, fixed in 3% paraformaldehyde, permeabilized with 0.1% Triton X-100 and finally blocked with 1% BSA for 30 min at room temperature. The cells were then incubated with AR rabbit polyclonal antibody diluted 1∶200 in blocking buffer for 1 h at RT. Finally, the cells were washed with PBS and incubated with FITC-labelled anti-rabbit secondary antibody diluted 1∶1000 in blocking buffer for 30 min at room temperature. The cells were then observed under a fluorescence microscope (Zeiss, Axiovert 25, Germany).

### Co-immunoprecipitation and Western Blot Analysis

The LNCaP cells were plated in 100 mm culture dishes and treated with conjugate or DHT for 24 h. The whole-cell lysates were prepared as described previously [26] in immunoprecipitation (IP) buffer containing 50 mM Tris-HCl (pH 8.0), 150 mM NaCl, 5 mM EDTA, and 1% Triton X-100. Protein aliquots of 500 μg were then incubated overnight at 4°C using 2 μg of antibody directed against AR. The Protein A-Cl Agarose beads were then added and further incubated for 6 h at 4°C. The immunoprecipitates were washed four times with the IP buffer and the immunocomplexes were recovered by boiling in SDS sample buffer. Finally the western blot was carried out using anti-AR, anti-SRC-1 and anti-GRIP-1 antibodies (Santa Cruz, CA, USA). For western blot analysis, the cell lysates were prepared after harvesting them in lysis buffer. About 40 μg of total protein was electrophoresed by 12% SDS-PAGE, and the western blotting was carried out using standard protocol. In brief, the analyzed proteins were transferred to nylon membranes. The blots were then blocked with TBST buffer (20 mM Tris-Cl, pH 7.5, 150 mM sodium chloride, 0.05% Tween-20) containing 5% skim milk powder. They were then washed with TBST buffer and incubated overnight at 4°C with the same buffer containing appropriate amounts of primary antibodies: caspase-3, Bax, Bcl-2, Akt, p-Akt, ERK, p-ERK, AR, SRC-1, GRIP-1 (1∶500) and β-actin (1∶1000). The blots were then washed and incubated with anti-rabbit secondary antibody (1∶20,000) conjugated with horseradish peroxidase (HRP). Color development was performed in the dark by using an ECL kit (Amersham, GE Healthcare, Buckinghamshire, UK). The developed blots were subjected to densitometric analysis by ImageJ 1.43 software (NIH, USA) using β-actin as internal control.

### Transfection

The LNCaP cells were grown and transiently transfected with pMMTV-neomycin-luciferase plasmid (300 ng/105 cells) in RPMI media supplemented with 10% FBS using polyfect transfection reagent (Qiagen, CA, USA) according to manufacturer’s instructions. Then, 24 h after transfection, the cell culture media were changed with media containing 5% charcoal-stripped FBS to reduce the contaminating steroids from the serum and incubated for an additional day before initiating the treatments. In case of PC-3 cells, they were similarly transfected, but in addition to pMMTV-neomycin-luciferase construct, they were also transfected with pSG5-hAR-puro plasmid (full length hAR in pSG5 expression vector) in the ratio of 5∶1 as indicated above. For experiments involving co-regulators, 50 ng of co-activator plasmids (25 ng each of GRIP-1 and SRC-1) along with pMMTV-neo-luc (250 ng) were transfected to LNCaP cells. To evaluate the transfection efficiency, 500 ng/105 cells of the SV40 promoter-Renilla luciferase (pRL-SV40) vector (Promega Madison, USA) were co-transfected as internal control. The cells were then treated either with vehicle or different concentrations (1–40 μM) of conjugate and/or 10 nM of DHT for 24 h, and the luciferase activity was measured according to the instructions provided in the kit (Promega, Madison, USA). Each experimental point was performed in triplicate and varied by less than 10%. The values of luciferase for each lysates were normalized to the Renilla luciferase activity.

For localization studies, pEGFP-AR plasmid was transfected to LNCaP and PC-3 cells (300 ng/105 cells). The cells were then incubated with 10 nM DHT with/without 10 and 20 μM conjugate and monitored regularly till 12 h under fluorescence microscope (Zeiss, Axiovert 25, Germany).

### siRNA Transfection

The siRNAs against human Akt and ERK and control siRNA were purchased commercially from Santa Cruz Biotechnology (USA). The LNCaP and PC-3 PCa cells were transfected with siRNAs (at a final concentration of 100 nM) using polyfect transfection reagent (Qiagen, CA, USA) according to manufacturer’s instructions. After 24 h of transfection, the cells were washed properly and replaced with fresh medium. The cells were then treated with 20 μM conjugate for 24 h, and finally protein lysates were prepared on completion of treatment. The levels of p-Akt, p-ERK and cleaved caspase-3 expressions were detected by Western blot analysis.

### Statistical Analysis

Data are expressed as mean ± SEM and statistically evaluated using one-way ANOVA followed by Bonferroni *post hoc* test using Graph Pad Prism 5.04 software (Graph Pad Software, San Diego, CA, USA). A *p*-value of less than 0.05 was considered to be statistically significant.

## Results

### Inhibition of Cell Proliferation by Conjugate

LNCaP (AR positive) and PC-3 (AR negative) cells were cultured with different concentrations of RESV, PTER and conjugate for 24 h in complete RPMI and DMEM media, respectively. Our data showed that the treatment of LNCaP and PC-3 PCa cells with RESV, PTER and conjugate resulted in a dose dependent inhibition of cell proliferation. As shown in Table 1, in case of LNCaP cells, the conjugate resulted in almost 50% reduction in the number of live cells at 40±1.12 μM while it was around 66.4±1.39 and 82.2±2.19 μM in case of PTER and RESV treated cells respectively after 24 h of treatment. Similarly, in case of PC-3 cells the IC_50_ value of conjugate, PTER and RESV was found to be 45±1.50, 75±2.55 and 95.0±1.13****μM respectively after 24 h treatment (Table 1). Furthermore, when tested in noncancer cell lines like CHO and COS-1, all the compounds were found to have IC_50_ values above 100 μM concentration. Thus both the cancer cell lines were found to be more sensitive to conjugate treatment as compared to PTER and RESV as depicted by the lower IC_50_ values in all of them. Further, our result showed that PTER-ITC conjugate is a potent cytotoxic agent in both AR positive and AR negative cell lines albeit to a marginally higher level in the former as compared to latter.

**Table 1:** Cytotoxicity induced by resveratrol, pterostilbene and conjugate in noncancer (COS-1, CHO) and prostate cancer (PC-3, LNCaP) cell lines after 24 h exposure as determined by MTT assay.

Cell lines	IC_50_ value^a^		
	Resveratrol (μM)^b^	Pterostilbene (μM)^b^	Conjugate (μM)^b^
PC-3	95.0±1.13	75±2.55	45±1.50^c^ ^d^
LNCaP	82.2±2.19	66.4±1.39	40±1.12^c^ ^d^
COS-1	>100 μM	>100 μM	>100 μM
CHO	>100 μM	>100 μM	>100 μM

a 50% growth inhibition as determined by MTT assay (24 h drug exposure).

b Compound tested in triplicate, data expressed as mean value ± SEM of three independent experiments.

c *p*<0.001 between resveratrol and conjugate treated group.

d *p*<0.001 between PTER and conjugate treated group.

### Differential Sensitivity of PC-3 and LNCaP Cells to Conjugate Induced Apoptosis

In milieu of the MTT assay results, we extended our study to examine whether PCa cells undergo apoptosis after conjugate treatment by using Annexin-V/PI double staining assay and flow cytometry analysis. After PCa cells were incubated with different concentrations of conjugate, the cells were stained with Alexa Fluor 488-conjugated Annexin-V and PI, which can assess the early and late apoptotic cell populations. As shown in Fig. 1B, the conjugate produced a dose-dependent increase in the apoptotic cell population in both the PCa cells studied. Treatment of PC-3 cells with increasing doses of conjugate for 24 h resulted in an increase in early apoptotic cells from about 44% to 68% and late apoptotic cells from 12% to 16% at 20 and 40 μM concentrations, as compared to vehicle treated groups respectively (Fig. 1B upper panel). Since PC-3 cells lack a functional p53 protein, it was of interest to determine whether the presence of wild type p53 affects cellular sensitivity to the cell death caused by conjugate because p53 is known to regulate apoptosis in different stimuli. We addressed this question by determining the sensitivity of LNCaP cells (wild type p53) towards conjugate-induced apoptosis by FACS analysis. Treatment of LNCaP cells for 24 h with increasing doses of conjugate resulted in a gradual increase of early apoptotic cells (Annexin V positive only) from 52% to 72% at 20 and 40 μM, respectively (Fig. 1B, lower panel). The late apoptotic cells (Annexin V and PI positive) also increased significantly from 8% to 18.5% (Fig. 1B, lower panel) as compared to only 0.23% of early apoptotic cells and almost negligible number of late apoptotic cells in the negative control group treated with 0.1% DMSO. The histogram in the right panel (Fig. 1C) indicates statistical analysis of three similar independent experiments for both the cell lines. Interestingly, the total number of apoptotic cells was statistically non-significant (*p*<0.05) between LNCaP and PC-3 cells when tested with different concentrations of conjugate, suggesting that p53 protein was not involved in the regulation of conjugate-induced apoptosis of PCa cells.

### Conjugate Induced Stage Specific Arrest of Prostate Cancer Cells

**Figure 2:**
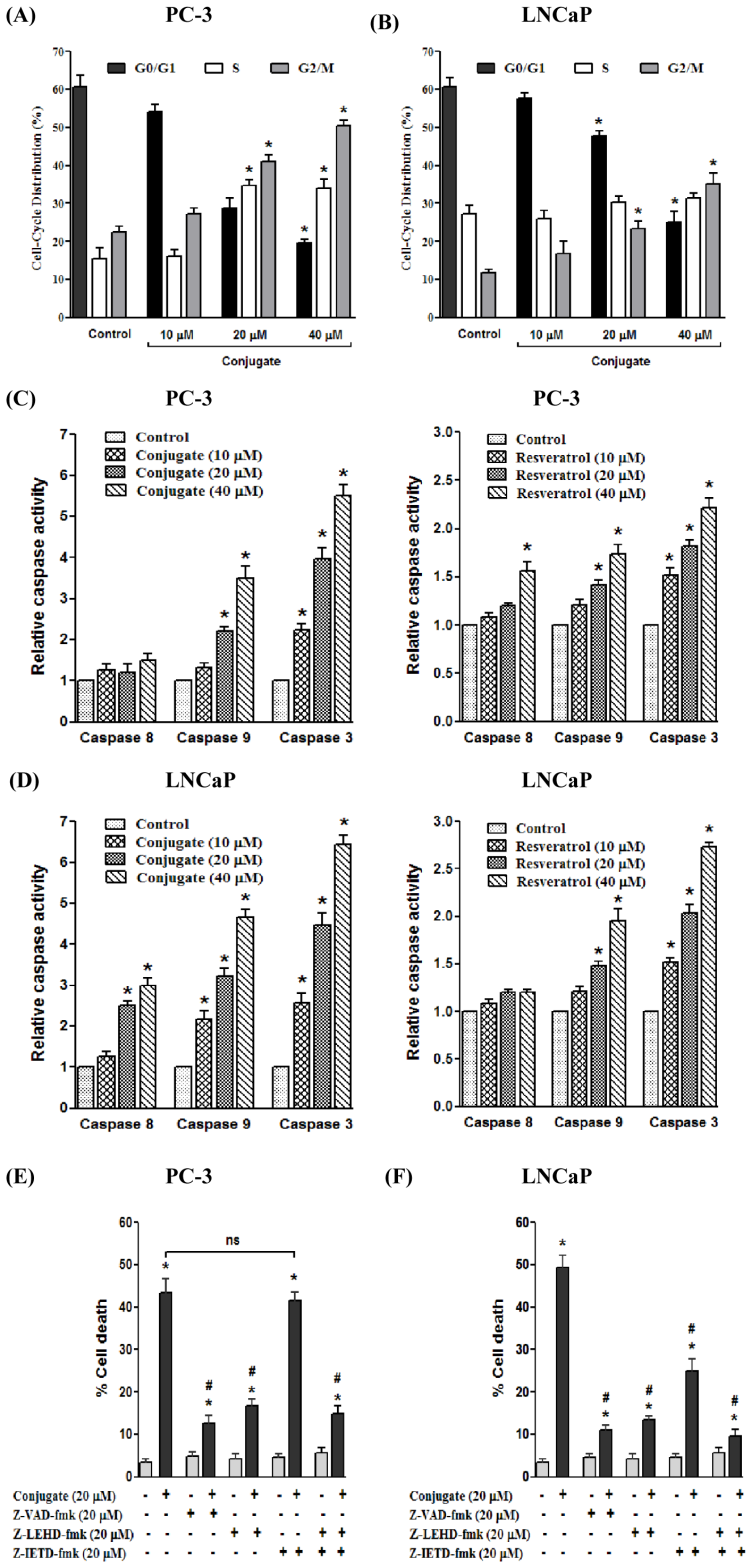
The conjugate induces G2/M phase arrest and caspase dependent cell death in prostate cancer cells. Cell cycle distribution of (A) PC-3 and (B) LNCaP cells upon treatment with varying doses of conjugate. Results are presented as mean ± SEM of three independent experiments. *represents statistically significant difference with respect to vehicle treated PC-3 and LNCaP cells respectively corresponding to each stage of cell cycle at *p*<0.05. (C) The effects of varying doses of conjugate (left panel) and resveratrol (right panel) on caspase-8, -9 and -3 activities in PC-3 and (D) LNCaP cells. Results are the mean ± SEM of three independent experiments. *represents statistically significant difference with respect to control cells for respective caspases tested at *p*<0.05. (E) The effect of caspase inhibitors on conjugate-induced cell death in PC-3 and (F) LNCaP cells. The cells were pre-treated with 20 μM of respective caspase inhibitors: Z-LEHDFMK (caspase-9 inhibitor); Z-IETDFMK (caspase-8 inhibitor); and Z-VAD-FMK (general caspase inhibitor) for 1 h before the addition of 20 μM conjugate. Cell death was measured 24 h after conjugate treatment using MTT assay. Data are the mean ± SEM of three independent experiments. *and # represents statistically significant difference with respect to control (vehicle) and conjugate treated cells respectively for each cell lines at *p*<0.05. ns, non-significant.

Based on the growth and DNA synthesis inhibitory responses of conjugate in PCa cells, we next examined its effect on cell cycle progression. As shown in Fig. 2A and 2B, treatment of PC-3 and LNCaP cells with increasing doses of conjugate for 24 h resulted in a dose dependent increase in the accumulation of cells in G2/M phase with concomitant decrease in G1 phase cells. In case of PC-3 cells, the effect observed at 40 μM conjugate was the greatest with approximately 50% of the cells being arrested in G2/M phase, compared to only 22% in control group (Fig. 2A). A similar trend in G2/M phase arrest was also demonstrated in LNCaP cells (35% in treated cells against 11% in control cells), although the total population of cells could not reach a high level of 50% as observed in PC-3 cells. As shown in Fig. 2B, the conjugate treatment resulted in an accumulation of 16–35% of cells in G2/M phase with increasing doses of conjugate. Thus, conjugate-mediated growth inhibition of both PC-3 and LNCaP cells correlated with G2/M phase cell cycle arrest.

### Conjugate Activates Caspase-3 via Caspase-9

Activation of both extrinsic and intrinsic caspase pathways has already been known to be the major mechanisms of apoptotic cell death in most cellular systems. To better understand the underlying cellular pathways for conjugate-induced death of PCa cells, a possible role of caspase in this process was investigated by measuring the activities of caspase-8, -9, and -3 in these tumor cells. While caspase-8 and caspase-9 are essential proteases of extrinsic and intrinsic apoptotic pathways respectively, caspase-3 acts as downstream effectors of both these pathways. Treatment of PC-3 cells with increasing doses (10, 20 and 40 μM) of conjugate and RESV for 24 h caused a dose-dependent augmentation in caspase-9 and caspase-3 enzyme activities. This increment was comparatively higher in case of conjugate treated cells as compared to cells treated with same dose of RESV (Fig. 2C). For caspase-8, although there was marginal increase in its activity at high dose of RESV treatment, no such induction was observed in case of conjugate treatment (Fig. 2C). On the contrary, the results obtained in case of LNCaP cells showed a significant increase in all the three caspase i.e. caspase-8, -9 and -3 on conjugate treatment indicative of involvement of both intrinsic (through caspase-9) and extrinsic (through caspase-8) pathways in apoptotic process by the conjugate in this cell line (Fig. 2D, left panel). Furthermore, our results indicated that activation of caspase-9 occurs prior to that of caspase-8 (at lower dose), which suggests that the mitochondrial pathway might be essential for conjugate-induced apoptosis. On the other hand, RESV treatment also showed significant increase in caspase -9 and -3 activities but to a lesser extent as compared to the conjugate (Fig. 2D, right panel) (*p*<0.05). Thus the above data clearly suggests that conjugate induced apoptosis in PC-3 cells is mediated via the intrinsic pathway, while both the intrinsic and extrinsic pathways contributes to apoptosis in LNCaP cells. Also the conjugate was found to be more effective than RESV in inducing apoptosis in both the PCa cell lines tested.

Furthermore, to elucidate the pathway which was predominant for conjugate-induced apoptosis, pharmacological inhibitors of specific caspases were employed to probe if they could protect the cells from undergoing apoptosis. As shown in Fig. 2E and 2F, Z-VAD-FMK, a general caspase inhibitor, showed significant inhibition of cell death in both PC-3 and LNCaP cells suggesting that apoptosis is the predominant form of cell death induced by the conjugate. In case of PC-3 cells, Z-LEHD-FMK, a specific inhibitor of caspase-9 also inhibited conjugate induced cell death by 73% while Z-IETD-FMK, a specific inhibitor of caspase-8, was completely ineffective in blocking conjugate induced cell death (*p*<0.05) (Fig. 2E). Furthermore, in case of LNCaP cells, the inhibitor of caspase-9 almost completely blocked the conjugate induced apoptosis while inhibitor of caspase-8 only partially inhibited it (Fig. 2F). The conjugate induced cell death was most prominently inhibited when the cells were pre-treated with both caspase-8 and caspase-9 inhibitors. Together, these data further strengthens our finding that conjugate induced apoptosis involves caspase -9/−8/−3 and caspase-9/−3 pathways in LNCaP and PC-3 cells respectively.

### Bcl-2 and Bax are Involved in Apoptosis by Conjugate

**Figure 3:**
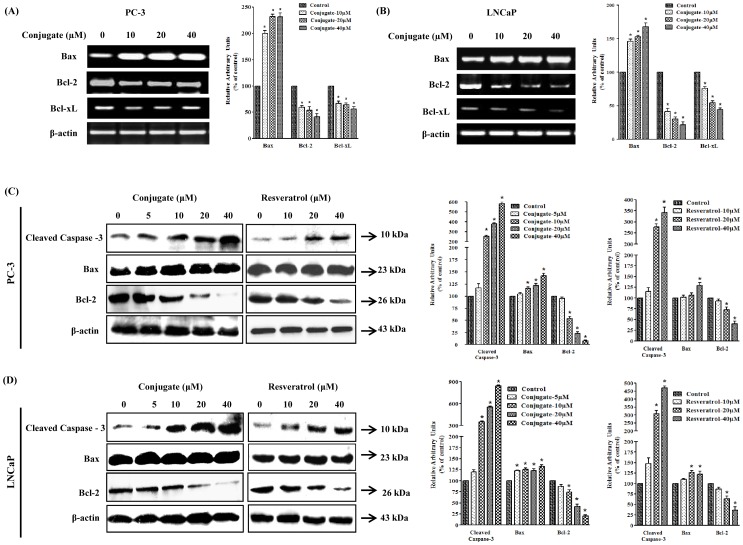
Transcriptional and translational analysis of various apoptotic marker genes in response to conjugate. Expression patterns of various apoptotic marker genes in response to varying doses of conjugate treatment as determined by RT-PCR in (A) PC-3 and (B) LNCaP cells. (C) Immunoblot analysis of various apoptotic marker genes in response to different doses of conjugate and resveratrol in PC-3 and (D) LNCaP cells. The histogram on the right panel of each figure represents densitometric analyses of the image data and expressed as percent of control in conjugate treated cells respectively where the results are mean ± SEM of three independent experiments. *represents statistically significant difference with respect to control for each genes at *p*<0.05.

Bcl-2 forms a heterodimeric complex with apoptotic Bax protein, thereby neutralizing its apoptotic effect. Therefore, the ratio of Bax/Bcl2 is often considered as a decisive factor in determining cell death or survival. In the present study, treatment of cells with conjugate resulted in a decrease in the expression of *Bcl-2* and *Bcl-xL* with a concomitant increase in *Bax* gene in both LNCaP and PC-3 cells (Fig. 3). This resulted in a substantial increase in Bax/Bcl2 ratio, which generally favors apoptosis. As shown in Fig. 3, with a concomitant increase in the level of Bax, there was a decrease in Bcl-2 and Bcl-xL levels for both PC-3 (Fig. 3A) and LNCaP (Fig. 3B) cells, respectively. The increase in Bax level by conjugate coincided with increase in caspase-3 activation, finally leading to apoptosis. It was found that the conjugate showed a marked enhancement of caspase-3 expression by about 8.5- and 6-folds in LNCaP (Fig. 3D) and PC-3 (Fig. 3C) cells, respectively at highest doses of conjugate treatments. Interestingly, although RESV treatment also significantly enhanced the levels of caspase-3 expression in both LNCaP (Fig. 3D) and PC-3 (Fig. 3C) cells (4.5- and 3.5-folds respectively) at its highest dose of treatment (40 μΜ), it was much lower than that caused by the conjugate at the same dose (*p*<0.05). Similar pattern of enhancement was also shown by RESV and conjugate for Bax in both LNCaP (Fig. 3D) and PC-3 (Fig. 3C) cells. These results suggest that the conjugate-induced apoptosis in PCa cells are partly through Bax-dependent pathway.

### Effect of Inhibitor of p53 on Conjugate Induced Apoptosis

**Figure 4:**
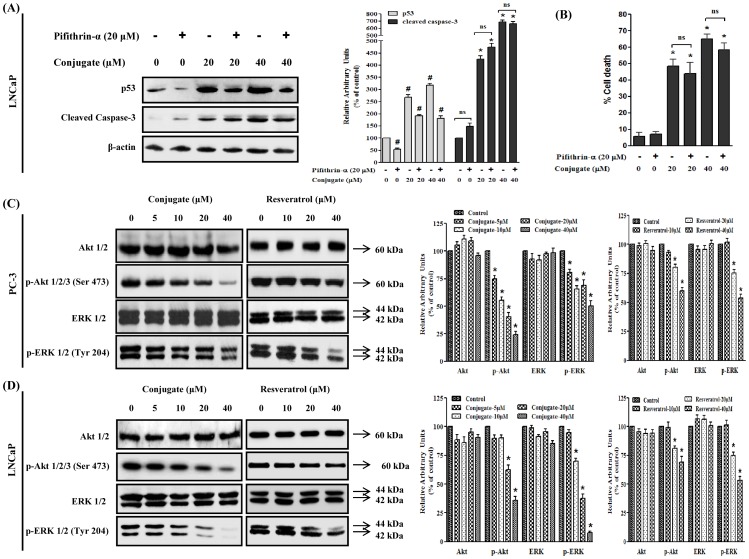
Role of p53 protein on conjugate induced cell death in LNCaP cells. Effect of p53 inhibitor (PFT-α) on (A) the expression of apoptotic genes as determined by immunoblot analysis and (B) cell death in conjugate treated LNCaP cells as determined by MTT assay. Data are the mean ± SEM of three independent experiments. # and *indicates significant difference with respect to the controls for p53 and caspsase-3 proteins respectively at *p*<0.05. (C) Immunoblot analysis to show the phosphorylation patterns of Akt and ERK in PC-3 and (D) LNCaP cells in response to varying doses of conjugate and resveratrol treatments. The histogram on the right panel of each figure represents densitometric analyses of the image data and expressed as percent of control where the results are mean ± SEM of three independent experiments. *represents statistically significant difference with respect to control for each group at *p*<0.05.

Since there was no significant difference in the number of apoptotic cells between conjugate treated LNCaP (wild type p53) and PC-3 (p53 null) cells (*p*<0.05), it was intriguing to check if p53 has any role in conjugate induced apoptosis of PCa cells. For this study we employed a pharmacological approach using specific wild type p53 inhibitor, pifithrin-α (PFT-α). PFT-α is a small molecule that binds to the DNA binding domain of p53, thereby inhibiting its transcriptional activity. For our experiment the LNCaP cells were pre-treated with 20 μM PFT-α for 2 h prior to the addition of 20 and 40 μM of conjugate for next 24 h. Subsequently the drug treated cells were used to check caspase-3 activity and cell death by immunoblot analysis (Fig. 4A) and MTT assay (Fig. 4B) respectively. As expected, PFT-α significantly down regulated the expression of p53 protein while conjugate treatment caused a dose dependent increase in its expression which, however, was significantly attenuated in presence of PFT-α (Fig. 4A). Further, the expression of cleaved caspase-3 in cells treated with combination of conjugate and PFT-α was almost similar to that of only conjugate treated cells suggesting that p53 was not involved in regulation of conjugate induced apoptosis. Next we determined the effect of p53-inhibition on conjugate induced cell death by using MTT assay. In agreement with the above findings the cell death resulting from a 24 h exposure to 20 and 40 μM conjugate did not differ significantly between LNCaP cells treated with or without PFT-α (Fig. 4B). Collectively, these results demonstrate that conjugate-induced apoptosis and inhibition of cell growth could not be related with the activation of p53 signaling pathway.

### Effects of Conjugate on Phosphorylation Status of Akt and ERK1/2

The Akt signaling pathway is one of the most critical pathways in regulating cell survival. Phosphorylation of Akt provides cells with a survival signal that allows them to withstand apoptotic stimuli. Similar to Akt, another signaling molecule, ERK1/2, also regulates the proliferation and differentiation of cells at various stages of cell cycle. It has been earlier reported that inhibition of ERK1/2 pathway could lead to the induction of apoptosis. To probe the involvement of Akt and ERK in regulating apoptosis induced by conjugate and RESV in LNCaP and PC-3 cells, we assessed the effect of conjugate and RESV on the level of phosphorylated Akt and ERK after 24 h of treatment. Our data showed that conjugate molecule significantly decreased the phosphorylation of Akt at Ser_473_ and ERK at Tyrosine_204_ in a dose-dependent manner in case of PC-3 cells (Fig. 4C). The level of p-Akt was decreased by almost 2.2- and 4.4-folds at 20 and 40 μM while the level of p-ERK was reduced to 1.3- and 1.7-folds at similar doses, respectively (Fig. 4C) (*p*<0.05). On the other hand, in case of RESV treatment the level of p-Akt decreased by about 1.2- and 1.6-folds at 20 and 40 μM while the level of p-ERK was reduced by 1.3 and 1.8-folds at similar doses, respectively. Furthermore, when tested in LNCaP cells, both RESV and conjugate exposure had negligible effects on the level of p-Akt below 20 μM, while in case of p-ERK, the inhibitory effect was dose-dependent as was observed in PC-3 cells and it was almost completely inhibited at highest dose of conjugate treatment (Fig. 4D) (*p*<0.05).

### Effect of Akt and ERK Gene Silencing on Conjugate-induced Apoptosis of PC-3 Cells

**Figure 5:**
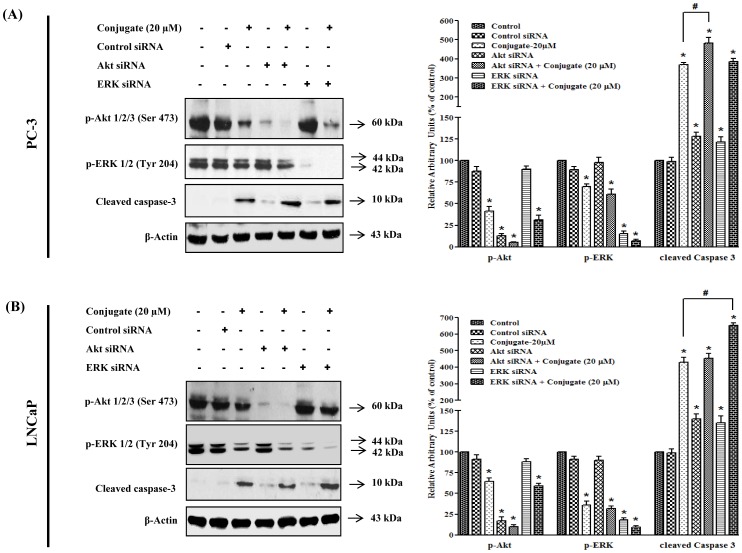
Differential role of PI3K/Akt and MAPK/ERK pathways in conjugate induced apoptosis of prostate cancer cells. Effects of siRNA mediated silencing of Akt and ERK on conjugate-induced apoptosis of (A) PC-3 and (B) LNCaP cells. LNCaP and PC-3 PCa cells were transfected with siRNAs (at a final concentration of 100 nM) using polyfect transfection reagent. After 24 h of transfection the cells were treated with 20 μM conjugate and allowed to grow for another 24 h. The cell lysates were prepared and the level of p-Akt, p-ERK and cleaved caspase-3 proteins were detected by immunoblot analysis. The histogram on the right panel of each figure represents densitometric analyses of the image data and expressed as percent of control where the results are mean ± SEM of three independent experiments. *and # represents statistically significant difference with respect to control and 20 μM conjugate treated groups respectively at *p*<0.05.

In order to confirm the role of Akt and ERK in conjugate-induced apoptosis, we silenced both Akt and ERK using their respective siRNAs and examined their effects on the regulation of expression of cleaved caspase-3. The results of siRNA induced silencing of Akt and ERK protein levels were confirmed by immunoblot analysis. As shown in Fig. 5A, transfection of PC-3 cells with Akt and ERK specific siRNAs (but not with control siRNA) resulted in approximately 80–90% reduction in the expressions of p-Akt and p-ERK. Treatment of PC-3 cells with 20 μM conjugate resulted in significant increase (3.7-folds) in cleaved caspase-3 protein expressions as compared to control which was enhanced further to 4.8-folds in presence of Akt siRNA (*p*<0.05). Since the combined effects of blockade of Akt by siRNA and conjugate were found to cause enhanced expression of cleaved caspase-3 as compared to their individual responses, it could be suggested that there is direct involvement of Akt dependent pathway in the conjugate induced apoptosis of PC-3 cells. Further, as a single agent the siRNA for ERK increased the expression of caspase-3 by 1.2-folds which was further increased to 3.9- folds in presence of conjugate. However, this increment in caspase-3 expression was similar to that of conjugate alone, suggesting that most probably the conjugate-induced cleavage of caspase-3 did not directly involve ERK pathway at least in PC-3 cells. A similar pattern of results were obtained when A6730 (Akt kinase inhibitor) and PD98059 (ERK inhibitor), were used for blocking Akt and ERK signaling pathways respectively (Fig. S1A). Collectively, the data suggested that the conjugate-induced apoptosis of PC-3 cells are mediated mainly through Akt-pathway.

### Effect of Akt and ERK Gene Silencing on Conjugate-induced Apoptosis of LNCaP Cells

To determine whether conjugate-induced apoptosis of LNCaP cells relates to Akt or ERK signaling pathways, the effect of inhibition of Akt and ERK on apoptosis was evaluated in the presence and absence of conjugate in LNCaP cells. As shown in Fig. 5B, Akt siRNA, as expected, completely abolished the phosphorylation of Akt and caused a 1.4-folds increase in cleaved caspase-3 protein level. When Akt-silenced LNCaP cells were treated with conjugate, there was about a 4.5-folds increase in cleaved caspase-3 protein level, which was almost comparable to increment caused by conjugate alone. This data suggested that, probably Akt pathway did not play any major role in inducing apoptosis in LNCaP cells. Further, ERK gene silencing showed increase in cleaved caspase-3 level (1.35-folds) when compared to vehicle treated control. ERK-silenced LNCaP cells when treated with conjugate, it significantly increased the level of cleaved caspase-3 by 6.5-folds, which was about 1.4 times higher than that induced by conjugate alone (*p*<0.05). This results were further validated using pharmacological inhibitors of Akt and ERK where co-treatment of LNCaP cells with PD98059 and conjugate significantly increased the level of cleaved caspase-3, while combining A6730 with the conjugate, did not show any significant increase as compared to only conjugate treated group (Fig. S1B). These results thus indicate that in contrary to PC3 cells, ERK played a key role in apoptosis of LNCaP cells by conjugate molecule while Akt inhibition alone was insufficient to induce apoptosis.

### Conjugate Inhibits Expression of AR

**Figure 6:**
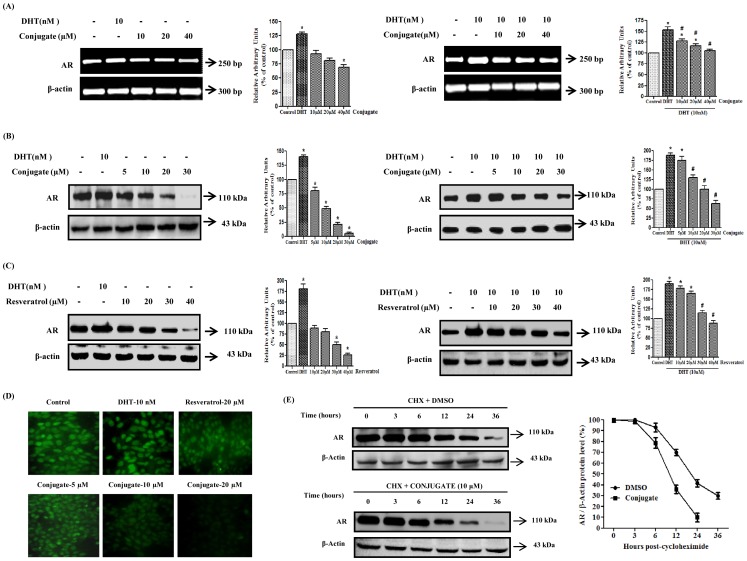
Effect of conjugate on androgen receptor expression in LNCaP cells. (A) Regulation of androgen receptor expression in LNCaP cells by varying doses of conjugate as determined by RT-PCR. (B) Immunoblot analysis to show the expression of androgen receptor in response to conjugate and (C) resveratrol. The histogram on the right panel of each figure represents densitometric analyses of the image data and expressed as percent of control where the results are mean ± SEM of three independent experiments. *and # represents statistically significant difference with respect to control and 10 nM DHT respectively at *p*<0.05. (D) Immunofluorescence analysis to show the expression of androgen receptor (400× magnification). (E) Effect of conjugate on androgen receptor protein turnover in LNCaP cells. LNCaP cells were grown to 60–70% confluency and treated with 10 μg/ml protein synthesis inhibitor cycloheximide (30 min pre-treatment) with or without 10 μM conjugate at 0 h. Cells were then harvested at various time points (0, 3, 6 12, 24 and 36 h) and lysates were prepared. Androgen receptor protein levels were determined by immunoblot analysis using anti-androgen receptor antibody and normalized to β-actin control. The results are representative of two independent experiments. CHX, cycloheximide.

Since AR plays a critical role in the initiation and progression of PCa, we detected the effects of conjugate on AR expression by reverse transcription-PCR and immunoblot analysis. For this, LNCaP cells, that express endogenous AR, were treated with varying concentrations of conjugate both in presence and absence of 10 nM DHT. As shown in Fig. 6A (left panel), the conjugate molecule had no significant effects on the expression of *AR* gene below 40 μM when treated in absence 10 nM DHT. However, interestingly, it demonstrated a dose-dependent inhibition of DHT (10 nM) induced AR transcription, which was reduced by about 1.2-, 1.3- and 1.4-fold (*p*<0.05) in response to 10, 20 and 40 μM of conjugates, respectively (Fig. 6A, right panel). Further, when we performed the immunoblot analysis of AR with increasing doses of RESV and conjugate treatments, we found a concomitant inhibition of the translation of this protein in response to various doses of the conjugate both in presence and absence of DHT (Fig. 6B). Treatment with 10 and 20 μM of conjugate yielded a significant inhibition of AR protein levels by 2- and 5-folds (*p*<0.05), respectively (Fig. 6B, left panel) while the same dose of RESV did not show any significant inhibition (Fig. 6C, left panel). In addition, the same dose of conjugate also inhibited DHT induced AR protein levels by 1.1- and 1.8-folds (Fig. 6B, right panel) while RESV did not show any significant inhibition till 20 μM (Fig. 6C, right panel) (*p*<0.05). Thus, the above data clearly suggested that both RESV and conjugate interferes with the translation of AR protein and interestingly, the latter was more potent than the former when tested at same concentrations.

In the next stage of experiment, immunofluorescence staining of LNCaP cells was performed in order to visualize the intensity of endogenous AR expression in this cell. As shown in Fig. 6D, treatment of cells with varying doses of conjugate resulted in almost complete abolition of fluorescent intensity in the cells, indicative of its anti-androgenic activity. As expected, DHT caused significant up regulation in the fluorescent intensity while 20 μM RESV showed marginal decrease in AR immunofluorescence which was in accordance to our earlier data.

### Conjugate Induced Decrease in AR Half-life

Since conjugate caused a significant dose-dependent decrease in AR protein level, we next focused our efforts on identifying whether post-translational modifications play any role in conjugate-mediated decrease in AR levels. To address this issue, LNCaP cells were treated with protein synthesis inhibitor, cycloheximide (50 μM), with/without 10 μM concentration of conjugate. At specified time points the cells were harvested and AR protein levels were measured by western blot analysis using anti-AR antibody. As shown in Fig. 6E, in conjugate-treated LNCaP cells, the half-life of AR protein was reduced from 20****h to 10 h as observed in the control cells treated with DMSO, suggesting that the observed decrease in AR protein level by conjugate could be due to post-translational degradation.

### Conjugate-induced Inhibition of AR-mediated Transcription of Luciferase Activity

**Figure 7:**
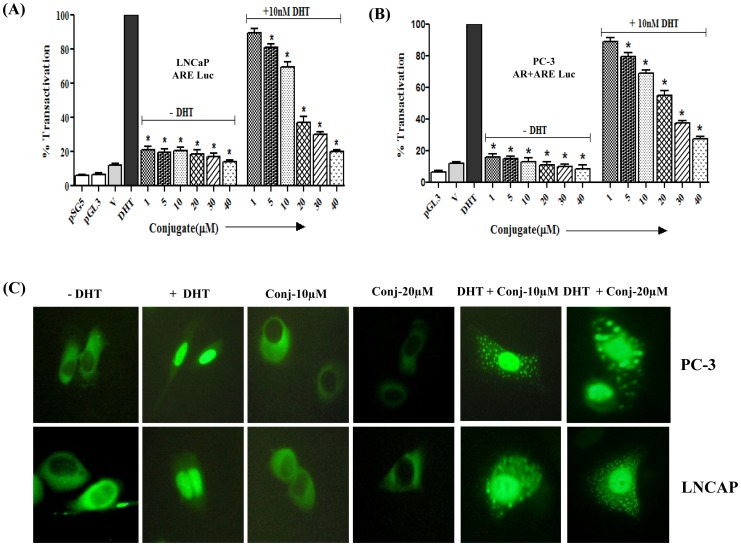
Conjugate inhibits androgen receptor transactivation and translocation in prostate cancer cells. Effect of conjugate on the transactivation of androgen receptor in presence/absence of 10 nM DHT in (A) androgen receptor positive LNCaP cells and (B) androgen receptor negative PC-3 cells. Luciferase activities are expressed as percentage of transactivation with respect to only 10 nM DHT treated group which is considered as 100%. *indicates statistically significant difference (*p*<0.05) with respect to DHT treated groups. (C) Effect of conjugate on the dynamics of nuclear translocation of androgen receptor as determined by green fluorescent protein (GFP)-androgen receptor construct in presence/absence of 10 nM DHT for 2 h.

In order to check the anti-androgenic activity of conjugate, AR positive LNCaP cells were transiently transfected with pMMTV-neomycin-luciferase construct. Ligand-activated AR binds to androgen response element (ARE) and its functional activation was tested using a luciferase reporter gene linked to MMTV promoter having multiple repeats of AREs. The effect of DHT (10 nM) on the luciferase activity was expressed as 100% transactivation. The conjugate alone (without DHT) did not induce transcriptional activation at any of the concentrations tested (1–40 μM). However, as shown in Fig. 7A, the conjugate exhibited a dose-dependent anti-androgenic activity by inhibiting the androgen-induced transactivation by about 73% at the highest dose tested (40 μM) as compared to only DHT treatment. In order to confirm the anti-androgenic activity of conjugate further, similar transactivation assay was also performed in AR negative PC-3 cells co-transfected with pSG5-hAR and pMMTV-neomycin-luciferase constructs. As shown in Fig. 7B, consistent with the results described above for LNCaP cells, in this cell line (PC-3) also the DHT-mediated luciferase activity was reduced by 19, 31, 63, 70 and maximum to 80% in the presence of 5, 10, 20, 30 and 40 μM of conjugate, respectively.

### Effect of Conjugate on the Nuclear Localization of AR

We next checked the effect of conjugate on the nuclear localization of AR in AR positive (LNCaP) and AR negative (PC-3) cells. Twenty-four hours after transient transfection of pEGFP-AR construct, both PCa cells were treated with 10 nM DHT in the presence and absence of conjugate (10 and 20 μM). Generally, any ligand for AR is expected to cause the nuclear localization of this receptor within a maximum period of 30 min. As shown in Fig. 7C, in the absence of androgen, the labeled AR protein was distributed in the cytoplasmic compartment, which in the presence of 10 nM DHT, was predominantly localized in the nucleus. Interestingly, the distribution of GFP-AR protein in the cells treated with conjugate alone was similar to that of vehicle-treated cells (without DHT), wherein the nuclear translocation of AR was inhibited at both the doses tested. However, as shown in Fig. 7C, when the cells are co-treated with DHT and conjugate, the nuclear localization of AR, as induced by DHT, was significantly inhibited by conjugate resulting in the dispersed distribution of AR proteins between the nuclear and the cytoplasmic compartments in both the cell lines. This pattern of distribution could be attributed to the anti-androgenic activity of the conjugate.

### The Conjugate Weakens the Interaction between AR and it Co-activators: SRC-1 and GRIP-1 in LNCaP Cells

**Figure 8:**
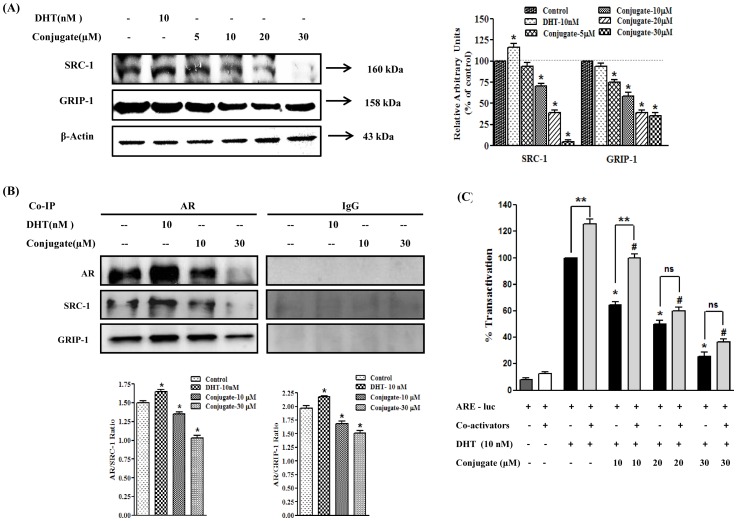
Conjugate weakens the interaction between androgen receptor and its co-activators. (A) Immunoblot analysis for the expression of androgen receptor co-regulators in response to conjugate in LNCaP cells and the histogram on the right panel of the figure represents densitometric analyses of the image data and expressed as percent of control in conjugate treated cells where the results are mean ± SEM of three independent experiments. *represents statistically significant difference with respect to control (*p*<0.05). (B) Effects of conjugate on the interactions of androgen receptor and its co-activators (SRC-1 and GRIP-1) as determined by co-immunoprecipitation analysis. Histogram in the lower panel represents ratio of androgen receptor to that of co-activators where results are mean ± SEM of three independent experiments. *represents statistically significant difference with respect to control (*p*<0.05). (C) Effect of conjugate on DHT induced transactivation of androgen receptor in presence/absence of co-regulators (SRC-1 and GRIP-1) in androgen receptor positive LNCaP cells. Luciferase activities are expressed as percentage of transactivation with respect to only DHT treated group which is considered as 100%. *and # indicates statistically significant difference from DHT treated cells in absence and presence of co-regulators respectively and **indicates significant level of difference between two adjacent groups with and without co-activator at *p*<0.05. ns, non-significant.

It is well documented that the intrinsic ligand-dependent activity of AR is potentiated through its interaction with co-regulators namely SRC-1 and GRIP-1. Since the conjugate repressed AR transcriptional activity and subsequently its transactivation, it is also probable that conjugate might suppress AR transactivation by interrupting the function of its co-activators. Hence, in the next stage alterations in the expression patterns of co-activators (GRIP-1 and SRC-1) were analyzed in response to DHT and conjugate treatment by immunoblot analysis. As shown in Fig. 8A, the expression of both the co-activators, GRIP-1 and SRC-1, were greatly reduced in LNCaP cells treated with conjugate. When treated with 10 and 20 μM of conjugate, the expression of SRC-1 protein was decreased by 1.4- and 2.6-folds (*p*<0.05) while the same dose reduced GRIP-1 level by 1.7- and 2.6-folds, respectively (*p*<0.05). As expected, DHT significantly increased the expression of SRC-1 (1.2-fold) (*p*<0.05), however, there was no significant change in the expression of GRIP-1.

Furthermore, to check if the conjugate interferes with the direct interaction of co-activators (SRC-1 and GRIP-1) to AR, co-immunoprecipitation analysis was performed in LNCaP cells, which has endogenous AR. As shown in Fig. 8B, lysates immunoprecipitated with AR antibody showed decreased band intensities for SRC-1 and GRIP-1 with increasing doses of conjugate, suggesting reduced interaction of AR-SRC-1 and AR-GRIP-1 in presence of conjugate. As expected, 10 nM DHT in turn showed enhanced interactions with those co-activators thus validating the co-immunoprecipitation assay. No detectable proteins were shown in immunocomplexes precipitated with normal IgG. Together, these data indicate that the conjugate-induced suppression of AR transactivation may not be only through the direct suppression of AR expression, but also by the inhibition of expression and their subsequent interaction with its co-activators like SRC-1 and GRIP-1.

Since the conjugate decreased DHT-mediated AR transactivation and further down-regulated expressions of its co-activators like SRC-1 and GRIP-1, it was intriguing to check whether the administration of exogenous GRIP-1 and SRC-1 could reverse the effects of conjugate on AR function and PCa cell growth. As shown in Fig. 8C, the transactivation of AR was significantly enhanced in the presence of co-activators in the cells treated with DHT (1.25-fold) (*p*<0.05), as compared to its responses in the absence of those co-activators. However, when the cells were treated with increasing doses of conjugate (10–30 μM), it caused strong inhibition of DHT-induced AR transactivation in the absence of these co-activators. Our data showed that although the expression of exogenous co-activators rescued the AR-inhibitory activity as caused by the conjugate at low dose of 10 μM (64% vs 102%), it failed to completely revert back this inhibitory effect at 20 and 30 μM concentrations of conjugate where the difference between normal cells (i.e., without co-activator transfected) and co-activator transfected cells was almost non-significant (Fig. 8C) (*p*<0.05). These results suggested that the conjugate-mediated reduction of AR activation may be due, at least in part, to a decrease in the expression of AR co-activators and their recruitment to the promoter of the AR target gene.

## Discussion

We have earlier reported that PTER is a potent anticancer molecule with multiple targets of action in breast and PCa cells [14], [27]. On the other hand, ITCs are naturally occurring small molecules that are formed from glucosinolate precursors of cruciferous vegetables. Numerous investigations have shown that naturally occurring ITC and their synthetic analogues retard or inhibit tumor cell growth, both *in vitro* and *in vivo* [22], [28]. The ability of ITC to inhibit tumorigenesis is dependent on its structure, the animal species, target tissues, and the specific carcinogen employed [29]. However, the existing literatures are somewhat ambiguous about the chemopreventive activity of ITC which suggests that not all of them are suitable for their use as chemopreventive agents [30]. Due to their electrophilic reactivity, some ITCs are able to form adducts with DNA and induce mutations and chromosomal aberrations [31]. PTER on the other hand has not yet been reported to have any genotoxic effects. Based on these reports we hypothesized that by combining PTER with ITC, the newly developed conjugate molecule could bypass some of their individual toxic effects and at the same time would complement each other for inhibition of PCa more efficiently. RESV, a naturally occurring polyphenol is closely related to PTER and has similar biological activity. The anti–prostate cancer potential of RESV has been well documented in many *in vitro* and *in vivo* studies and a large amount of evidence suggests that it may be a promising molecule in both PCa treatment and prevention (reviewed in [32], [33]). Hence in the present study, we focused on the characterization of PTER-ITC conjugate, a hybrid molecule from PTER and ITC, for its efficacy as a potent apoptotic and anti-androgenic agent for PCa chemotherapy while directly comparing it to RESV. It is noteworthy to mention here that the response shown by conjugate in this study was much better than that of RESV. The probable reasons for the superior inhibitory effect of conjugate as compared to RESV on PCa cells may be (i) due to the higher binding affinity of the former to same target signaling proteins as that of the latter and or alternatively (ii) the conjugate and RESV may altogether have different protein targets due to differences in their chemical structures.

PCa proceeds through two distinct operationally divisible stages: androgen-dependent and androgen-independent. Therefore, in the present study, two different cell lines, LNCaP (androgen dependent) and PC-3 (androgen independent) were used to study the effect of this conjugate on PCa cells growth and AR regulation. The cell proliferation assay showed that the conjugate molecule caused a dose dependent cytotoxicity in both the cell lines. This cytotoxicity was more or less equally effective in suppressing proliferation of both PC-3 and LNCaP cells. These observations have clinical implications since the majority of human PCa at the time of diagnosis represent androgen-dependent as well as androgen-independent situations. The p53 tumor suppressor protein plays an important role in the regulation of apoptosis by different stimuli [34], [35]. Both p53-dependent and p53-independent apoptosis processes are known to occur in cells due to various insults. To delineate if the conjugate-induced apoptosis in PCa cells are p53-dependent, we compared cancer cell vulnerability against conjugate in p53 positive LNCaP cells. Our functional study showed that the inhibition of p53 by its inhibitor (PFT-α) had no significant effects on the cell death caused by this conjugate, suggesting that the conjugate induced apoptosis of PCa cells are independent of p53. Further, the flow cytometry analysis showed that the conjugate molecule arrested the PCa cells in G2/M phase, suggesting that the conjugate-induced inhibition of cell proliferation involved both cell cycle arrest and apoptosis. During cell cycle, the G2/M checkpoint is a potential target for cancer therapy. It prevents DNA-damaged cells from entering mitosis and allows their repair of DNA that was damaged in late S or G2 phases prior to mitosis [36]. Data presented herein indicated that 10 and 20 μM concentrations of conjugate effectively inhibited proliferation of PC-3 and LNCaP cells by inducing apoptosis and causing cell cycle arrest. Our data are consistent with the results of previous cellular studies using other ITC analogues, such as sulforaphane and phenethyl isothiocyanate, where apoptosis induction, cell cycle arrest and/or molecular changes associated with growth inhibition were observed at a concentration of 50 μM or lower [22], [24].

Previous studies have shown that Akt and ERK-MAPK signaling pathways function cooperatively to promote prostate tumorigenicity and androgen independency [37]. The Akt pathway can be activated by various growth factors and plays a crucial role in promoting growth and blocking apoptosis in various cancer models including PCa [38]. Although the precise anti-apoptotic effects of Akt are still not very well understood, it has already been reported that activated Akt can phosphorylate several apoptosis-regulating proteins including BAD, a member of pro-apoptotic Bcl-2 family [39], [40]. BAD promotes cell death by interacting with anti-apoptotic Bcl-2 members such as Bcl-xL, which allows the multidomain pro-apoptotic Bcl-2 family members like Bax and Bak to aggregate and cause release of apoptogenic molecules (e.g., cytochrome c) from mitochondria to the cytosol culminating into caspase activation and cell death [41]. In the present study, it could be speculated that the conjugate induced inactivation of Akt by decreasing the level of phosphorylated-Akt in a concentration-dependent manner and further reduction in the levels of Bcl-2, Bcl-xL and concomitant increase in the expressions of Bax followed by caspase-9 and caspase-3, as a whole, contributed to the promotion of apoptosis in these two cell lines. Survival-signaling cascade in many cells involves PI-3-kinase, Akt, and also cross-communication between PI-3-kinase and ERKs [42]. Since sustained activation of ERK1/2 is necessary for cell survival and proliferation [42], suppression of ERK activation by any agent can mediate apoptosis, which may be linked to subsequent inhibition of Bcl-2 in PCa cells [43], [44]. The current data is in accordance to the previous report where it was speculated that the inhibition of p-ERK1/2 and Bcl-2 by conjugate may be another pathway for apoptosis in PCa cells [43], [44]. In conclusion, the current data suggests the involvement of at least two different protein kinase pathways for regulating induction of apoptosis by the conjugate in these two different PCa cells.

The AR is a key regulator in the development and growth of PCa and current therapeutic strategies utilizes anti-androgens that prevent AR activation and/or disrupt endogenous androgen production. However, several reports showed that these therapies ultimately fail as a result of AR activation by non-steroidal physiological signals as well as the existence of mutant ARs in PCa cells that can be activated by non-androgenic steroids and certain growth factors [45]–[47]. Thus, there is an urgent need for testing new therapies based on different mechanisms to target AR signaling for androgen-dependent PCa. Treatment that aims at reducing AR expression may represent an attractive approach to target androgen signaling in PCa. Our results demonstrated that the conjugate disrupts androgen signaling at multiple stages of AR signaling pathways including its transcription, translation and degradation as a part of its growth arrest program in LNCaP cells. Thus, it could be presumed that the conjugate is one of the few compounds along with RESV [48], tea polyphenol EGCG [49], luteolin [50], andrographolide [51], emodin [52] and indole-3-carbinol [53], which also disrupts AR cellular activities by down regulating its transcription as well as translation.

The transactivation function of AR can be regulated by several co-regulators (co-activators and co-repressors) [54], [55]. These proteins do not directly bind to DNA and are recruited to the promoter regions of the AR-target genes through protein-protein interactions with AR. Therefore, a decrease in the expression levels of AR co-activators or the interruption of their interaction with AR in PCa cells could contribute to inhibition of AR signaling and ultimately the growth of PCa cells [56]. The first identified member of the co-activator family that regulated steroid receptor action was SRC-1 [57], which is functional in many different tissue types and enhances transcriptional activity of AR in a ligand-dependent manner [8]. Co-regulators such as GRIP-1 and SRC-1 interact with AR to enhance ligand-dependent transactivation of AR. The expression of SRC-1 and GRIP-1 increases in cancer and recurrent PCa after medical or surgical castration [58], suggesting that GRIP-1 and SRC-1 may be involved in the development and progression of PCa. In this connection, it is worth mentioning here that the conjugate significantly decreased the expression of both SRC-1 and GRIP-1 and also interrupted the interaction of AR with SRC-1 and GRIP-1 as shown by co-immunoprecipitation analysis. Further, transfection with exogenous SRC-1 and GRIP-1 also could not restore the inhibition caused by the conjugate at higher doses, suggesting that some other co-regulators may also be involved in conjugate-induced decrease in AR regulation. From the above results, it could be hypothesized that most probably, not only the conjugate has anti-AR activity due to its receptor antagonizing properties, but it might also inhibit some other interacting proteins (mainly co-activators like SRC and GRIP-1), which in turn regulates the AR transcriptional activity.

Finally we checked if the conjugate has any effect on the localization of AR to the nucleus. Our data showed that the conjugate at higher doses (10–20 μM) prominently inhibited the nuclear localization of AR after 2 h incubation with it. A similar pattern of inhibition was also reported in COS-1 cells where the nuclear-localized fluorescently labeled AR decreased after incubation with 40 μM emodin for 2 h (an anthraquinone derivative isolated mainly from the root and rhizome of *Rheum palmatum)* [52]. However, in case of conjugate and DHT co-treatments, a prominent cytoplasmic fluorescence was observed with a decrease in nuclear intensity. This may be either due to inhibition of nuclear import of AR or augmentation of nuclear export of AR. A similar pattern of inhibition was reported in case of RESV where the nuclear-localized AR decreased after incubation with 10 μM RESV for 24 h, but not for 3 h [48]. This result was further validated by electrophoretic mobility shift assay where the conjugate treatments decreased the binding activity of AR to its cognate response element of *PSA* gene (data not shown). However, based on these data it is difficult to confirm if this is mainly due the inhibition of nuclear translocation of the activated AR or a physical interference of AR association with the ARE through modulation of co-regulators other than SRC-1 and GRIP-1. Further detailed analysis in this regards are warranted to specifically designate the anti-androgenic effect of this conjugate using in vitro and in vivo AR-knockin/knockout animal models.

## Conclusions

**Figure 9:**
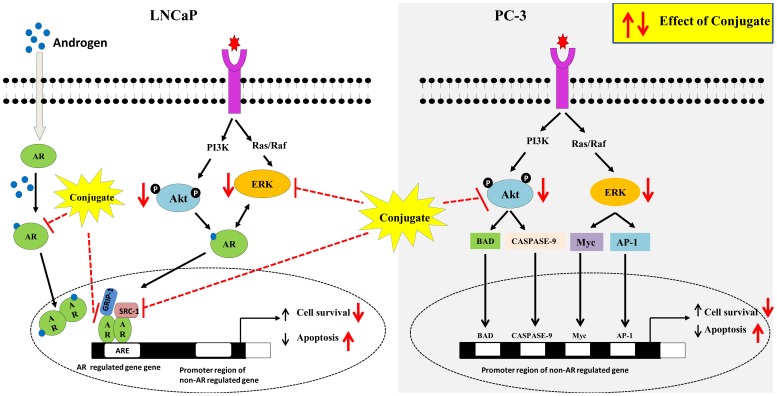
Proposed scheme for conjugate mediated actions on LNCaP and PC-3 prostate cancer cells. Conjugate inhibits both androgen receptor and ERK signalling and finally contributing to its downstream effects of decreased cell viability and increased apoptosis in androgen receptor positive LNCaP cells. In case of androgen receptor negative PC-3 cells, inhibition of Akt and its downstream targets contributes to conjugate mediated decrease in cell viability and increased apoptosis.

Based on the above findings, it could be concluded that PTER-ITC conjugate inhibits the AR-regulated pathways in PCa cells involving various signaling cascades as shown in Fig. 9. The conjugate significantly inhibited cell proliferation, induced apoptosis, cell cycle arrest, down regulated the expression of AR and abrogated DHT induced activation in PCa cells. Our findings further support the differential involvement of these protein kinase pathways (Akt and ERK) in regulating apoptosis induction by conjugates in these two different PCa cells. Moreover, results on steroid sex hormones are consistent with PTER-induced inhibition of PCa cells as shown by Wang et al. [16]. Hence the current data provides a new set of information which states that the PTER-ITC conjugate inhibits androgen activity on LNCaP cells. To the best of our knowledge, this is the first study to show that PTER-ITC conjugate acts as AR antagonist not only by regulating its expression, but also by preventing its entry into the nucleus followed by recruitment of co-activators. Thus, our results suggest that a novel strategy of combining PTER and ITC may benefit patients with PCa. However, further in depth research including animal experimentation on prostate tumor models are needed in order to fully understand the inhibition of tumor progression and/or treatment of PCa and other human malignancies with this compound before considering it for clinical trials.

## Supporting Information

Figure S1 (A) Effects of the Akt Kinase inhibitor (A6730) and/or the ERK inhibitor (PD98059) on the conjugate-induced apoptosis of PC-3 and (B) LNCaP cells. The cells were pre-treated with A6730 (5 μM) and/or PD98059 (20 μM) for 1 h before the addition of 20 μM conjugate for additional 24 h (total inhibitor exposure time was 25 h). The collected cell lysates were then immunoblotted using respective antibodies. The histogram on the right panel of each figure represents densitometric analyses of the image data and expressed as percent of control where the results are mean ± SEM of three independent experiments. *and # represents statistically significant difference with respect to control and 20 μΜ conjugate treated groups respectively at *p*<0.05.
(TIF)

Table S1 **Primers sequences used for RT-PCR reactions.**
(DOC)
