# Supplementary figures and images for: Pterostilbene-Isothiocyanate Conjugate Suppresses Growth of Prostate Cancer Cells Irrespective of Androgen Receptor Status
Source: PLoS One. 2014 Apr 3;9(4):e93335. doi: 10.1371/journal.pone.0093335 (PMC3974779; doi:10.1371/journal.pone.0093335)

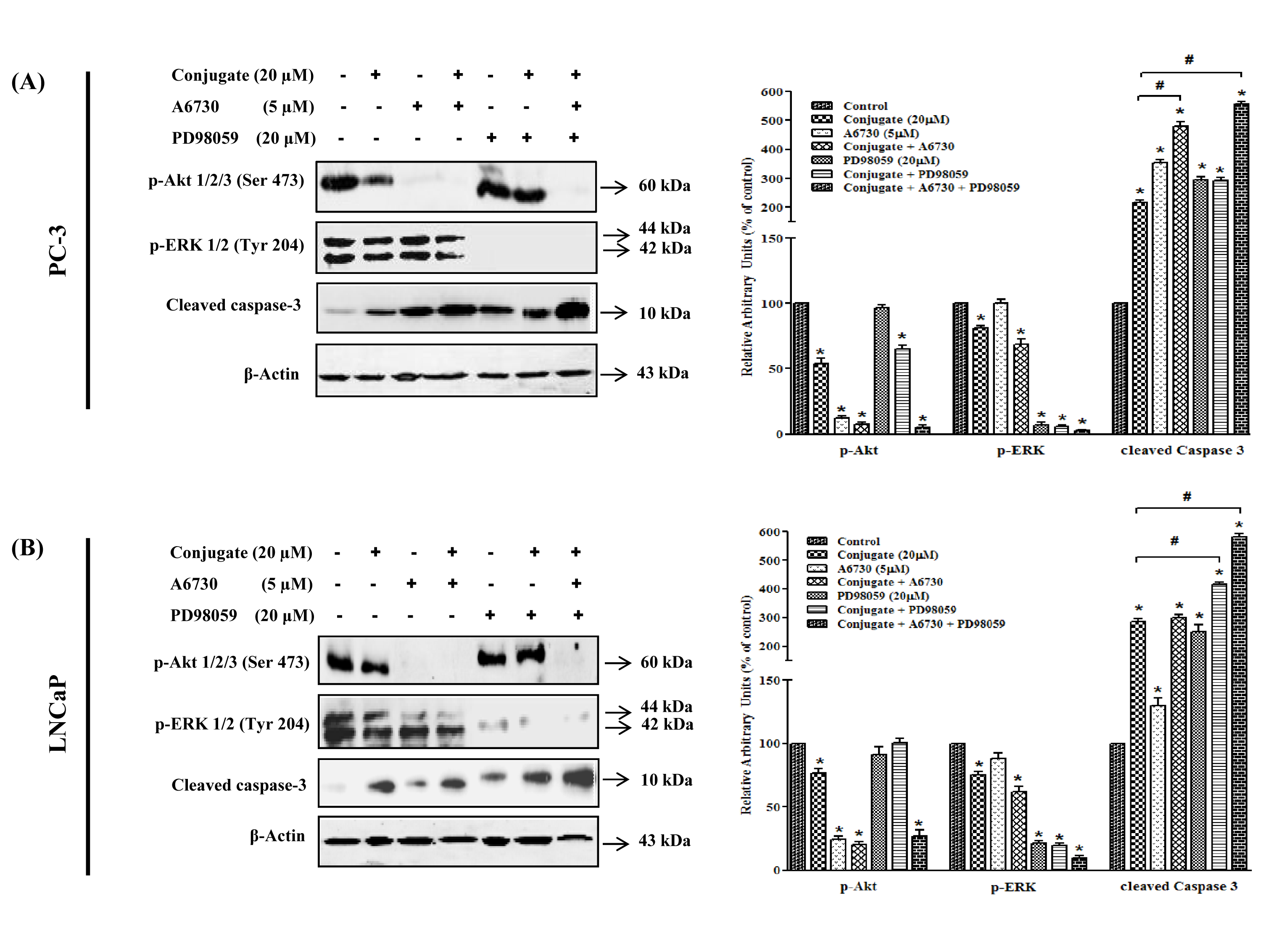

Supplement: Figure S1 — (A) Effects of the Akt Kinase inhibitor (A6730) and/or the ERK inhibitor (PD98059) on the conjugate-induced apoptosis of PC-3 and (B) LNCaP cells. The cells were pre-treated with A6730 (5 μM) and/or PD98059 (20 μM) for 1 h before the addition of 20 μM conjugate for additional 24 h (total inhibitor exposure time was 25 h). The collected cell lysates were then immunoblotted using respective antibodies. The histogram on the right panel of each figure represents densitometric analyses of the image data and expressed as percent of control where the results are mean ± SEM of three independent experiments. *and # represents statistically significant difference with respect to control and 20 μΜ conjugate treated groups respectively at p<0.05. (TIF) [file pone.0093335.s001.tif]
