# Supplementary material for: Pterostilbene-Isothiocyanate Conjugate Suppresses Growth of Prostate Cancer Cells Irrespective of Androgen Receptor Status
Source: PLoS One. 2014 Apr 3;9(4):e93335. doi: 10.1371/journal.pone.0093335 (PMC3974779; doi:10.1371/journal.pone.0093335)
Supplement: Table S1 — Primers sequences used for RT-PCR reactions. (DOC) [file pone.0093335.s002.doc]

| **Gene** | **Primer Sequence (5’-3’)** | **Product size (bp)** | **Cycles used** | **Annealing temp. (oc)** |
| --- | --- | --- | --- | --- |
| Bcl2 (F)  Bcl2 (R) | CGACTTTGCAGAGATGTCCA  ATGCCGGTTCAGGTACTCAG | 148 | 25 | 58 |
| Bax (F)  Bax (R) | TGCAGAGGATGATTGCTGAC  GAGGACTCCAGCCACAAAGA | 317 | 25 | 60 |
| Bcl-xL (F)  Bcl-xL (R) | AACTCTTCCGGGATGGGGTAA  AATTCTGAGGCCAAGGGAACT | 200 | 25 | 60 |
| AR (F)  AR (R) | TCCATCTTGTCGTCTTCGGAA  GGGCTGGTTGTTGTCGTCT | 250 | 25 | 59 |
| β-Actin (F)  β-Actin (R) | TCACCCACACTGTGCCCCATCTACGA  CAGCGGAACCGCTCATTGCCAATGG | 300 | 20 | 57 |
|  |  |  |  |  |

**Table S1**
